# Ferulic Acid Alleviates Oxidative Stress-Induced Cardiomyocyte Injury by the Regulation of miR-499-5p/*p*21 Signal Cascade

**DOI:** 10.1155/2021/1921457

**Published:** 2021-12-07

**Authors:** Shenghui Sun, Yang Ruan, Mingjing Yan, Kun Xu, Yao Yang, Tao Shen, Zening Jin

**Affiliations:** ^1^The Key Laboratory of Geriatrics, Beijing Institute of Geriatrics, Institute of Geriatric Medicine, Chinese Academy of Medical Sciences, Beijing Hospital/National Center of Gerontology of National Health Commission, Beijing 100730, China; ^2^Cardiac and Macrovascular Disease Center, Beijing Tiantan Hospital, Capital Medical University, Beijing 100070, China; ^3^Peking University Fifth School of Clinical Medicine, Beijing 100730, China

## Abstract

**Objective:**

To investigate the protective effects and regulatory mechanisms of ferulic acid on oxidative stress-induced cardiomyocyte injury.

**Methods:**

We established a cardiomyocyte oxidative stress cell model by H_2_O_2_ treatment and a mouse heart injury model by isoprenaline infusion of male C57BL/6 mice. Ferulic acid was applied to treat oxidative stress-induced cardiomyocyte injury. DHE staining was used to detect ROS production. DNA fragmentation, TUNEL assay, and cleaved caspase-3 were used to analyze cell apoptosis. Real-time PCR and Western blotting were used to analyze miRNA and protein levels to investigate the regulatory mechanisms of ferulic acid on oxidative stress-induced cardiomyocyte injury.

**Results:**

Ferulic acid pretreatment significantly inhibited H_2_O_2_- and isoprenaline-induced oxidative stress and cell apoptosis by promoting miR-499-5p expression and inhibiting p21 expression. MiR-499-5p inhibition reversed the protective effects of ferulic acid. Further study found that ferulic acid could also attenuate isoprenaline-induced mouse heart fibrosis and cell apoptosis by reducing oxidative stress, inflammation, and apoptosis in vivo.

**Conclusions:**

We proved that ferulic acid protects cardiomyocytes from oxidative stress-induced injury by regulating the miR-499-5p/p21signaling pathway, which provides insight into the clinical application of ferulic acid in the treatment of cardiovascular diseases.

## 1. Introduction

Cardiovascular disease (CVD) is the main cause of death worldwide. With the increase in the aging population, the mortality rate caused by cardiovascular disease is on the rise [1], with more than 17 million people dying every year worldwide. Death and disability caused by cardiovascular disease also cause serious public health and economic burdens [2]. Therefore, the prevention and treatment of cardiovascular diseases are worldwide priorities.

Oxidative stress, defined as excessive production of reactive oxygen species (ROS) and a disturbance between ROS and antioxidants, has been shown to cause cumulative damage and to play an important role in the development of cardiovascular diseases, such as heart failure, heart infarction and ischemia, and heart remodeling [3]. Many studies have shown that oxidative stress is increased in cardiomyocyte injury and heart failure. Excessive ROS production can cause cell dysfunction, protein and lipid peroxidation, and DNA damage, which may lead to irreversible cell damage and death and a variety of cardiovascular diseases [3]. Many cardiovascular diseases can lead to mitochondrial function impairment or loss in cardiomyocytes and cause a chronic increase in ROS production and retention of antioxidant function, which leads to catastrophic positive feedback of mitochondrial DNA (mtDNA) damage and more ROS generation [4]. Excessive ROS production can directly damage the expression and function of key proteins that are involved in the excitation-contraction coupling of cardiomyocytes and the activation of a variety of proinflammatory signal kinases and transcription factors, which lead to cardiomyocyte injury and apoptosis [5]. Cardiomyocyte death leads to reduced heart function and heart failure. ROS can also stimulate cardiac fibroblast proliferation and matrix metalloproteinase activation, leading to increases in extracellular matrix and heart remodeling [5]. Therefore, it is very important to find new drugs to inhibit oxidative stress for the treatment of cardiovascular diseases.

Previous studies have found that many compounds in plant extracts, such as resveratrol, curcumin, anthocyanins, and ginsenosides, have cardiovascular regulation functions. These plant-derived polyphenol compounds provide a new choice for the treatment of cardiovascular diseases, and their applications are more effective and cost-effective and have fewer side effects. With the development of new research methods and the discovery of new compounds derived from natural plants, in-depth research on the mechanism of plant extracts provides an important basis for the development of new treatments for cardiovascular diseases.

Ferulic acid ([E]-3-[4-hydroxy-3-methoxy-phenyl] prop-2-enoic acid), one of the most common cinnamic acid derivatives, is a phenolic compound that is abundantly present in grains, fruits, vegetables, and herbs (such as rice, wheat, oranges, tomatoes, resina ferulae, and angelica) [6,7]. Ferulic acid is characterized by antioxidant properties, and its benzene ring contains hydroxyl and methoxy groups with antioxidant activity, which prevents cell injury and inflammatory reactions caused by free radicals. For example, ferulic acid can inhibit melanin production, enhance angiogenesis, promote wound healing, and delay the skin photoaging process. Ferulic acid can bind to phospholipids of biological membranes, protect cell membrane integrity and function, and alleviate the damage caused by free radicals. Ferulic acid has a low molecular weight and high bioavailability to penetrate cells [6,8]. Ferulic acid also has anti-inflammatory effects in vivo and in vitro [9]. Recent studies have also found that ferulic acid can protect cardiomyocytes from diabetes mellitus and doxorubicin-induced cardiac tissue injury in vivo [10–12]. However, its cardioprotective mechanisms remain unclear.

In this study, we investigated the protective effects and mechanisms of ferulic acid in H_2_O_2_- or isoprenaline-induced cardiomyocyte injury and provided novel insight into how ferulic acid regulates cardiomyocyte miR-499-5p and p21 expression in vivo and in vitro. These findings provide insights into the protective mechanisms of ferulic acid in cardiotoxicity and a new potential treatment method for oxidative stress-induced cardiovascular diseases.

## 2. Materials and Methods

### 2.1. Reagents

Ferulic acid was purchased from Sigma–Aldrich Co., Ltd. (St. Louis, MO, USA) and Solarbio Co., Ltd. (Beijing, China). P21, phospho-p38, p38, Bcl2, Bax, cleaved-caspase-3 (c-caspase-3), and GAPDH antibodies were purchased from Cell Signaling Technology (MA, USA). Horseradish peroxidase- (HRP-) conjugated secondary antibody was obtained from Cell Signaling Technology (MA, USA). Unless otherwise specified, all other chemicals were purchased from Sigma (St. Louis, MO, USA) or Solarbio Co., Ltd. (Beijing, China).

### 2.2. Cell Cultures and Treatments

H9c2 cells were cultured in Dulbecco's modified Eagle's medium (DMEM; Sigma, USA) containing 10% fetal bovine serum (FBS; HyClone, USA; v/v), 2 mmol/L glutamine, and antibiotics (10 mol/L penicillin G and 10 mol/L streptomycin) at 37°C in an incubator with 5% CO_2_. Before every experiment, the cells were incubated in DMEM without FBS for 24 hours. Ferulic acid was added in the medium 4 hrs before 200 *μ*M of H_2_O_2_ treatment. After 24 hrs of H_2_O_2_ treatment, the H9c2 cells were harvested for further analysis [13,14].

### 2.3. Assessment of Cell Viability

Cell viability was analyzed by MTT assay according to the manufacturer's instructions. H9c2 cells were seeded into 96-well plates. All samples were analyzed in triplicate. 3-[4, 5-Dimethylthiazol-2-yl]-2, 5-diphenyltetrazolium bromide (0.5 mg/ml) was added to the plates, and the cells were cultured for 4 hours. Samples were then analyzed by measuring the absorbance at 490 nm. The MTT assay kit was obtained from Solarbio Co., Ltd. (Beijing, China).

### 2.4. In Situ Detection of Reactive Oxygen Species (ROS)

ROS production was detected using dihydroethidium (DHE, Sigma), a fluorescent probe for detecting intracellular superoxide anion levels. H9c2 cells were stained with 10 *μ*M DHE for 30 min in a dark and humid chamber at 37°C to analyze ROS production in situ. The production of ROS was indicated using red fluorescence, observed by fluorescence microscopy, and subsequently quantified using ImageJ software (NIH) [13].

### 2.5. DNA Fragmentation Analysis (DNA Laddering)

After the treatment, the cells were lysed in lysate buffer containing 10 mM Tris-Cl (pH 8.0), 150 mM NaCl, 10 mM EDTA, 0.4% SDS, and 100 g/ml protease K. The cells were incubated at 37°C overnight with gentle agitation and extracted with phenol/CHCl3/isoamyl alcohol, followed by CHCl3/isoamyl alcohol. DNA fragmentation was detected by loading 10–20 µg of total DNA onto a 2% agarose gel in Tris-acetate/EDTA buffer and visualized by ethidium bromide staining as described previously [14].

### 2.6. TUNEL and Hoechst33342 Staining

TUNEL staining was performed in fixed cells or heart cryosections using a cell death detection kit from Roche, and the nuclei were then counterstained with10 mM Hoechst 33342 as previously described [15]. The nuclei were observed by fluorescence microscopy.

### 2.7. Transfection

Normal mimic control (NC), miR-499-5p mimics (miR-499-5p-m), miRNA inhibitor negative control (NCi), and miR-499-5p inhibitor (miR-499-5p-i) were chemically synthesized by GenePharma Co. (Suzhou, China) (Table 1). When the H9c2 cells reached 70% confluence, miRNA or siRNA was transfected using Lipofectamine RNAiMAX Transfection Reagent (Invitrogen, Carlsbad, CA, USA) according to the manufacturer's protocol. After 8 hours of transfection, the medium was replaced with fresh medium. Then, the cells were cultured for 24 hrs before H_2_O_2_ treatment [15].

### 2.8. Mouse Model of Isoprenaline-Induced Heart Injury Model and Oral Administration of Ferulic Acid

All animal experiments were approved by the Beijing Normal University, Animal Use and Care Committee, and the Guide for the Care and Use of Laboratory Animals (NIH publication #85–23, revised in 1996). Ten- to 12-week-old male C57BL/6J mice were randomly divided into the following four groups: sham + saline, isoprenaline + saline (ISO + saline), isoprenaline + ferulic acid (ISO + FA), and sham + ferulic acid (sham + FA). The mice were anesthetized with 1–1.5% isoflurane in oxygen. An osmotic pump (ALZET, Cupertino, CA; DURECT, Cupertino, CA) filled with isoprenaline (30 mg/kg per day) in saline or saline alone was delivered by infusion for 14 days after the operation to subcutaneously insert the pump [16]. Ferulic acid was dissolved in saline, and equal volumes of a freshly prepared ferulic acid solution or saline were administered to mice every day through oral gavage starting 3 days before the operation to the end of the experiments. The dosages of ferulic acid were 30 mg/kg/day, as reported previously [6]. All mice were identically housed and fed the same chow. Fourteen days after isoprenaline infusion, mice were anesthetized with 3–4% isoflurane and euthanized by cervical dislocation. The hearts were removed and weighed, and the ratio of the heart to the tibia length was analyzed. The heart tissues were kept in liquid nitrogen for further analysis.

### 2.9. H&E Staining and Sirius Red Staining

H&E staining was performed on adult mouse heart paraffin sections with a hematoxylin-eosin/H&E staining kit from Solarbio Co. (Beijing, China) as described previously [13]. Heart fibrosis was analyzed with the Sirius Red staining kit from Solarbio Co. (Beijing, China) according to the manufacturer's protocol. The collagen volume fraction was determined by performing quantitative morphometry as described previously [13].

### 2.10. RNA Extraction and Quantitative Real-Time Polymerase Chain Reaction (RT–PCR)

The expression of miR-499-5p was detected by RT–PCR. Briefly, total RNA was extracted with a TRIzol kit (Sigma) from H9c2 cells or mouse heart tissue following the manufacturer's protocol. Then, reverse transcription of the RNAs into cDNAs was performed with a First Strand cDNA Synthesis Kit (New England Biolabs). Then, real-time PCR was performed in a QuantStudio3 Real-Time PCR system (Thermo Fisher Scientific, USA) with a reaction mixture containing SYBR Green (Roche Applied Science, Mannheim, Germany). The reaction conditions for reverse transcription were as follows: 95°C for 30 s, 40 cycles at 95°C for 30 s, 60°C for 30 s, and 72°C for 30 s, as previously described [17]. The primers for miR-499-5p and U6 are listed in Table 1. U6 was used for normalization. The relative expression of miR-499-5p was calculated using 2^−ΔΔCt^, and the data are the average of 4–6 independent experiments.

### 2.11. Western Blotting

Western blotting was performed on cells or homogenized myocardial tissue using standard procedures with specific antibodies (p21, phospho-p38, p38, Bcl2, Bax, c-caspase-3, and GAPDH). All bands were visualized using chemiluminescence reagent (Perkin Elmer, Wellesley, MA, USA). GAPDH was used as the protein loading control. ImageJ software (NIH) was used to perform densitometric analyses (http://rsb.info.nih.gov/ij/) as reported previously [17].

### 2.12. Statistical Analysis

Data are expressed as the mean ± SEM of at least three experiments. Statistical analysis was performed using the statistical software GraphPad Prism 6.0 (GraphPad Software, CA, USA). Student's *t*-test was used to evaluate the differences between the two groups. Differences between multiple groups were analyzed by one-way ANOVA, followed by Bonferroni's procedure for multiple-group comparisons. *P* < 0.05 was considered statistically significant.

## 3. Results

### 3.1. Ferulic Acid Protects Cardiomyocytes from H_2_O_2_-Induced Cell Injury by Inhibiting ROS Production

**Figure 1:**
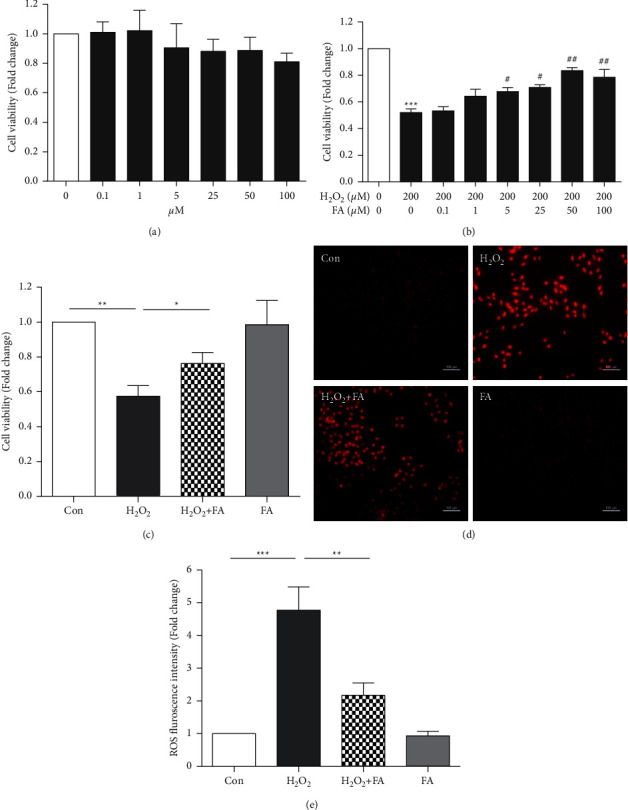
Ferulic acid protects cardiomyocytes from H_2_O_2_-induced cell injury by attenuating oxidative stress. (a) H9c2 cells were incubated with 0–100 *μ*M ferulic acid for 24 hrs, and the cell viability was analyzed by MTT assay (*n* = 4). (b) H9c2 cells were treated with 0–100 *μ*M ferulic acid and 200 *μ*M H_2_O_2_ for 24 hrs, and the cell viability was determined by the MTT assay (*n* = 5). (c) The viability of the cells in the control (Con), 200 *μ*M H_2_O_2_ (H_2_O_2_), 200 *μ*M H_2_O_2_ + 50 *μ*M ferulic acid (H_2_O_2_ + FA), and 50 *μ*M ferulic acid (FA) groups (*n* = 4). (d), (e) Reactive oxygen species (ROS) analysis by DHE staining in the Con-, H_2_O_2_-, H_2_O_2_ + FA-, and FA-treated cells (*n* = 4). ^∗^*P* < 0.05 vs. control, ^∗∗^*P* < 0.01 vs. control, ^∗∗∗^*P* < 0.001 vs. control, ^#^*P* < 0.05 vs. H_2_O_2_, and ^##^*P* < 0.01 vs. H_2_O_2_. FA indicates ferulic acid.

The cytotoxicity of ferulic acid was analyzed by MTT assay in H9c2 cells treated with different concentrations of ferulic acid (0, 0.1, 1, 5, 25, 50, and 100 *μ*M) for 24 hrs. We found that ferulic acid did not have significant cytotoxic effects in H9c2 cells at concentrations of 0–100 µM (Figure 1(a)). To evaluate the potential protective effects of ferulic acid, we established an oxidative stress-induced cell injury model by treating H9c2 cells with 200 *μ*M H_2_O_2_. We found that 200 *μ*M H_2_O_2_ markedly decreased viability (Figures 1(b) and 1(c)) and increased the ROS content in H9c2 cells (Figures 1(d) and.  1(e)). Next, H9c2 cells were pretreated with different concentrations of ferulic acid for 4 hrs and then treated with 200 *μ*M H_2_O_2_. Pretreatment with ferulic acid could increase cell viability in a concentration-dependent manner in the H_2_O_2_-induced H9c2 cell injury model. When the concentration of ferulic acid reached 50 *μ*M, the cell viability peaked (Figure 1(b)). Therefore, we used 50 *μ*M ferulic acid to perform the following experiments.

### 3.2. Ferulic Acid Attenuates H_2_O_2_-Induced Cell Injury by Inhibiting Cell Apoptosis in Vitro

**Figure 2:**
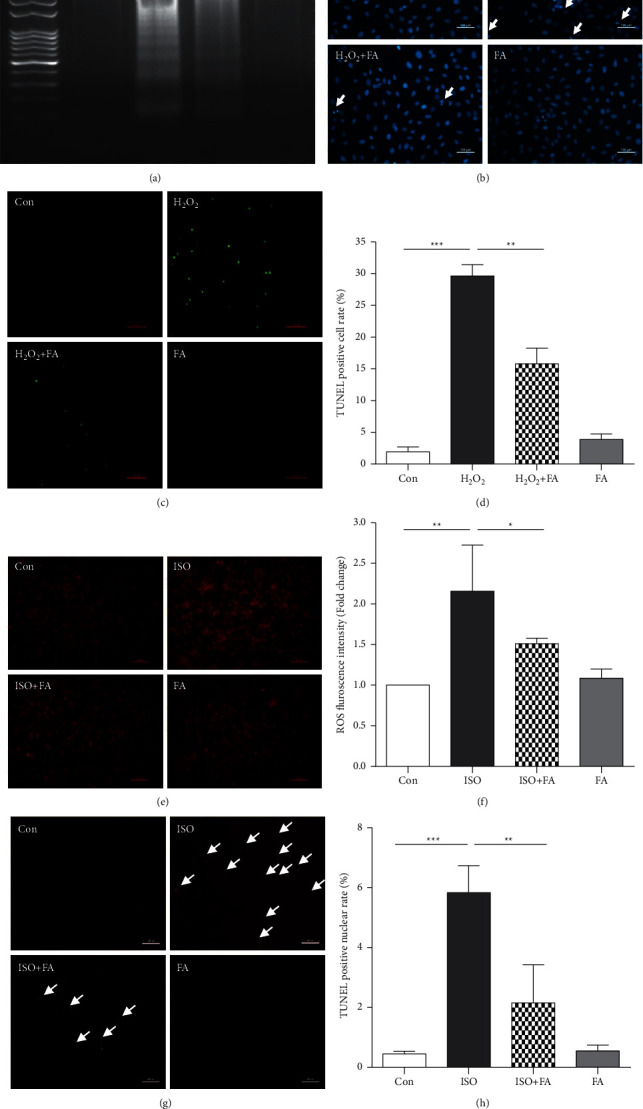
Ferulic acid attenuates H_2_O_2_-induced cell apoptosis in vitro. H9c2 cells were pretreated with 50 *μ*M ferulic acid for 4 hrs and then incubated with 200 *μ*M H_2_O_2_ for 24 hrs. (a) A representative image of DNA fragments analysis (DNA laddering) of Con-, H_2_O_2_-, H_2_O_2_ + FA-, and FA-treated cells. (b) Cell apoptosis was analyzed by Hoechst 33342 staining in the Con, H_2_O_2_, H_2_O_2_ + FA, and FA groups (*n* = 4). (c). (d) Representative TUNEL staining photos and the averaged data from the Con-, H_2_O_2_-, H_2_O_2_ + FA-, and FA-treated H9c2 cells (*n* = 4). (e), (f) Representative DHE staining photos and the averaged data from the Con, 10 *μ*M isoproterenol (ISO), 10 *μ*M isoproterenol + 50 *μ*M ferulic acid (ISO + FA), and FA groups (*n* = 4). (g). (h) Representative TUNEL staining photos and the averaged data from the Con, ISO, ISO + FA, and FA groups (*n* = 4). ^∗^*P* < 0.05, ^∗∗^*P* < 0.01, and ^∗∗∗^*P* < 0.001.

H_2_O_2_ (200 *μ*M) could induce cell apoptosis and resulted in ladder-like bands of DNA, confirming that H_2_O_2_ could cause apoptosis in H9c2 cells. Pretreatment with ferulic acid significantly reduced cell apoptosis in vitro (Figure 2(a)). Next, we analyzed nuclear morphology in control, H_2_O_2_-, H_2_O_2_ + FA-, and FA-treated cells. Hoechst 33342 staining showed that H9c2 cells exhibited typical chromatin condensation and increased apoptotic bodies after 24 hrs of H_2_O_2_ treatment. However, ferulic acid pretreatment could significantly decrease the chromatin condensation and apoptotic bodies in H_2_O_2_-treated H9c2 cells (Figure 2(b)). TUNEL staining also confirmed the antiapoptotic effect of ferulic acid in H_2_O_2_-treated H9c2 cells (Figure 2(c) and 2(d)). We observed a similar protective phenotype in a 10 *μ*M isoprenaline- (ISO-) induced H9c2 cell oxidative stress model in H9c2 cells by DHE staining (Figures 2(e) and 2(f)) and TUNEL staining (Figures 2(g) and 2(h)).

### 3.3. Ferulic Acid Attenuates H_2_O_2_-Induced H9c2 Cell Inflammation and Apoptosis by Regulating the miR-499-5p/*p*21 Signaling Cascade

**Figure 3:**
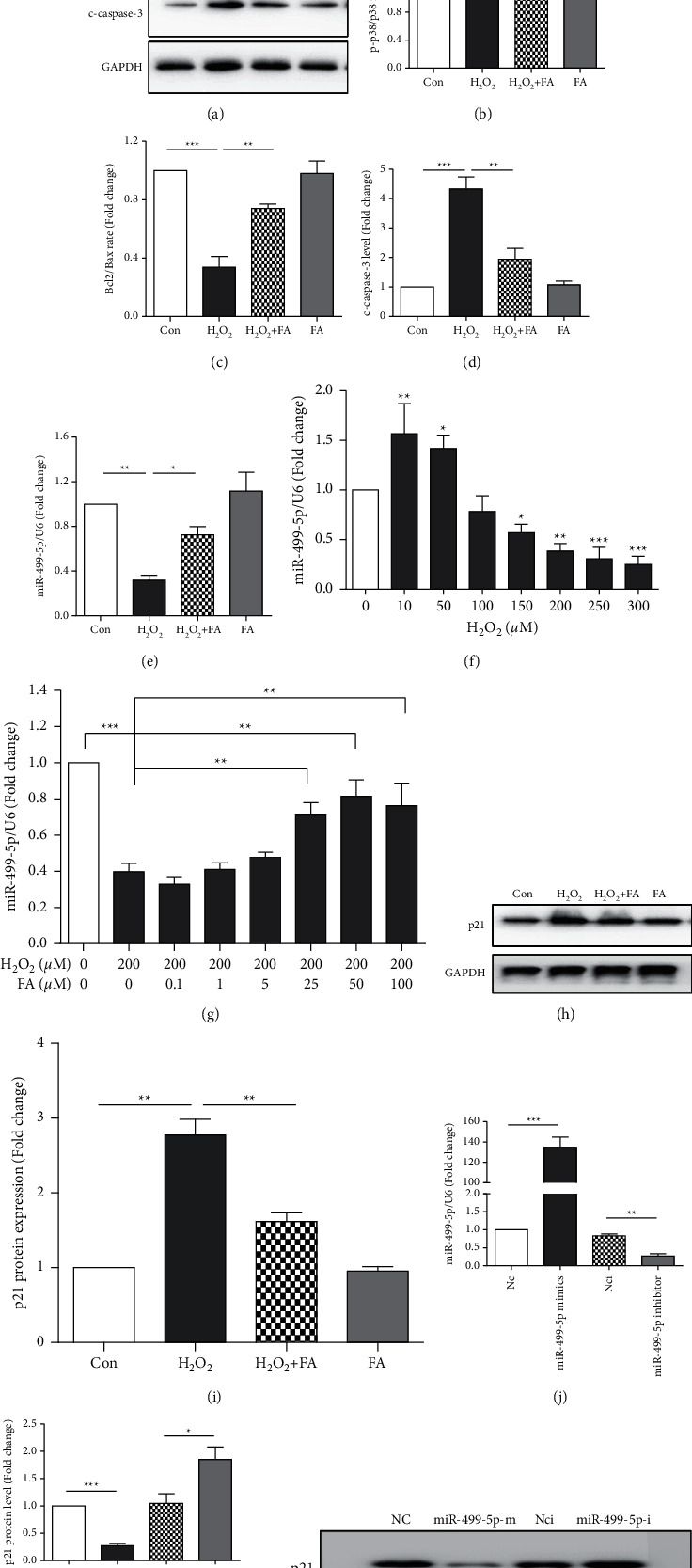
Ferulic acid protected cardiomyocyte from H_2_O_2_-induced cell injury by regulating the miR-499-5p/*p*21 signaling pathway. H9c2 cells were pretreated with 50 *μ*M ferulic acid for 4 hrs and then incubated with 200 *μ*M H_2_O_2_ for 24 hrs. (a), (b), (c). and (d) Western blot and the average protein level data of p-p38, p38, Bcl-2, Bax, and c-caspase-3 in the Con, H_2_O_2_, H_2_O_2_ + FA, and FA groups. GAPDH was used as the protein loading control (cropped blots,*n* = 4) (cropped blots, *n* = 4). (e) The miR-499-5p levels were analyzed by real-time PCR in the Con, H_2_O_2_, H_2_O_2_ + FA, and FA groups (*n* = 4). (f) The miR-499-5p levels were analyzed by real-time PCR after 0–300 *μ*M H_2_O_2_ treatment for 24 hrs (*n* = 4). (g) H9c2 cells were treated with 0–100 *μ*M ferulic acid and with/without 200 *μ*M H_2_O_2_ treatment for 24 (h) and miR-499-5p was determined by real-time PCR (*n* = 5). (h), (i) Western blot and the average data of the miR-499-5p target protein p21 in Con, H_2_O_2_, H_2_O_2_ + FA, and FA groups. GAPDH was used as the protein loading control (cropped blots, *n* = 4). (j) miR-499-5p levels in miRNA negative control (Nc), miR-499-5p mimics, miRNA inhibitor negative control (Nci), and miR-499-5p inhibitor-transfected H9c2 cells (*n* = 3). (k). (l) Western blot and the average data of the protein level of p21 in miRNA negative control (Nc), miR-499-5p mimics (miR-499-5p-m), miRNA inhibitor negative control (Nci), and miR-499-5p inhibitor (miR-499-5p-i)-transfected H9c2 cells (*n* = 3). ^∗^*P* < 0.05, ^∗∗^*P* < 0.01, and ^∗∗∗^*P* < 0.001.

Our data showed that H_2_O_2_-induced cell inflammation and apoptosis increased expression of proinflammatory proteins, such as Bax, phosphorylatedp38 (p-p38), and the active form of caspase-3 (cleaved caspase-3, c-caspase-3), and decreased the level of Bcl2 (Figures 3(a)–3(d)). Interestingly, pretreatment with ferulic acid inhibited proinflammation and proapoptotic signal activation after H_2_O_2_ treatment.

To investigate the regulatory mechanisms of the protective effect of ferulic acid, we screened several important miRNAs in cardiomyocytes. Interestingly, we found that miR-499-5p expression was decreased significantly in H_2_O_2_-treated H9c2 cells, and its expression was increased most significantly in ferulic acid-treated cells, which suggested that miR-499-5p might be involved in the protective effects of ferulic acid (Figure 3(e)). Next, we analyzed the expression of miR-499-5p after 0–300 *μ*M H_2_O_2_ treatment (Figure 3(f)). The results showed that 10–50 *μ*M H_2_O_2_ treatment increased the expression of miR-499-5p, while 150–300 *μ*M H_2_O_2_ treatment decreased miR-499-5p expression in a concentration-dependent manner (Figure 3(f)). These data suggest that miR-499-5p may be an oxidative stress response miRNA in cardiomyocytes. Interestingly, ferulic acid also increased miR-499-5p expression in 200 *μ*M H_2_O_2_-treated H9c2 cells in a concentration-dependent manner (Figure 3(g)). The proapoptotic protein p21, a downstream target gene of miR-499-5p, was negatively correlated with miR-499-5p levels in H9c2 cells (Figures 3(h) and 3(i)). We also used miR-499-5p mimics and inhibitors to overexpress/knock down miR-499-5p in H9c2 cells (Figure 3(j)). The p21 protein expression levels were significantly inhibited by the miR-499-5 mimic and increased by the miR-499-5p inhibitor, which validated the regulation of p21 by miR-499-5p reported previously (Figures 3(k) and 3(l)).

### 3.4. MiR-499-5p Is a Key Regulator of the Protective Effect of Ferulic Acid in Oxidative Stress-Induced Cardiomyocyte Injury

**Figure 4:**
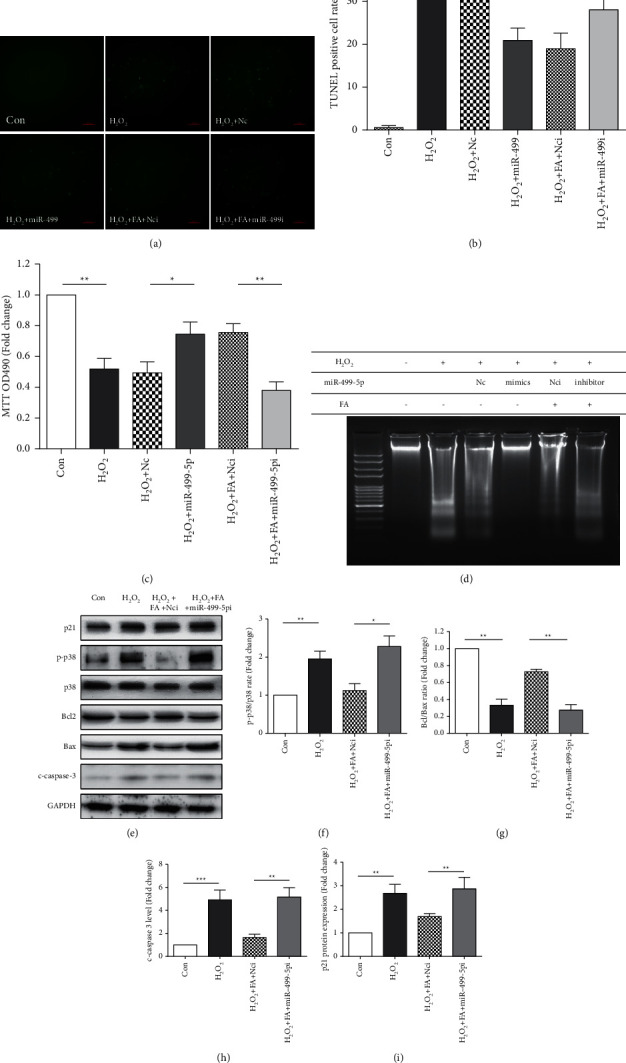
MiR-499-5p is a key regulator of the protective effect of ferulic acid in oxidative stress-induced cardiomyocyte injury. (a), (b) Cell apoptosis was analyzed by TUNEL staining in the Con, H_2_O_2_, and H_2_O_2_ + miRNA negative control (H_2_O_2_ + Nc), H_2_O_2_ + miR-499-5p mimics (H_2_O_2_ + miR-499-5p), H_2_O_2_ + ferulic acid + miRNA inhibitor negative control (H_2_O_2_ + FA + Nci), and H_2_O_2_ + ferulic acid + miR-499-5p inhibitor (H_2_O_2_ + FA + miR-499-5pi) groups (*n* = 4). (c) Cell viability was analyzed in the Con, H_2_O_2_, H_2_O_2_ + Nc, H_2_O_2_ + miR-499-5p, H_2_O_2_ + FA + Nci, and H_2_O_2_ + FA + miR-499-5pi groups by MTT assay (*n* = 4). (d) Cell apoptosis was detected by DNA laddering of Con, H_2_O_2_, H_2_O_2_ + Nc, H_2_O_2_ + miR-499-5p, H_2_O_2_ + FA + Nci, and H_2_O_2_ + FA + miR-499-5pi group cells. (e), (f), (g), (h), and (i) Western blot and the average protein levels data of p21, phosphorylatedp38 (p-p38), p38, Bcl-2, Bax, and c-caspase-3 in the Con, H_2_O_2_, H_2_O_2_ + FA + Nci, and H_2_O_2_ + FA + miR-499-5pi groups. GAPDH was used as the protein loading control. Cropped blots, *n* = 4. ^∗^*P* < 0.05, ^∗∗^*P* < 0.01, and ^∗∗∗^*P* < 0.001.

We investigated the function of miR-499-5p by overexpressing or inhibiting its expression in H9c2 cells. Overexpression of miR-499-5p by miR-499-5p mimic inhibited H_2_O_2_-induced cardiomyocyte apoptosis. Moreover, the miR-499-5p inhibitor reversed the protective effect of ferulic acid in H_2_O_2_-treated H9c2 cells (Figures 4(a)-4(d)). TUNEL, MTT, and DNA laddering assays also proved that miR-499-5p overexpression could inhibit H_2_O_2_-induced cell apoptosis, and miR-499-5p inhibitor could fully block the protective effect of ferulic acid on H_2_O_2_-treated H9c2 cells (Figures 4(a)-4(d)). Therefore, our data suggested that ferulic acid could protect cardiomyocytes from H_2_O_2_-induced injury by upregulating miR-499-5p.

To further investigate the protective effect of ferulic acid on H_2_O_2_-treated cardiomyocytes, we analyzed the expression levels of different proteins in cardiomyocytes upon miR-499-5p inhibitor transfection by Western blotting. The results showed that H_2_O_2_ led to a significant increase in p21 expression compared with the control group. As shown in Figures 4(e)-4(i), the expression of p21 in the H_2_O_2_ + FA + miR-499-5p inhibitor group was higher than that in the H_2_O_2_ + FA + Nci group, indicating that miR-499-5p could inhibit H_2_O_2_-induced cardiomyocyte injury by inhibiting p21 expression.

Interestingly, when p21 expression was upregulated due to the inhibition of miR-499-5p, the protective effects of ferulic acid on H_2_O_2_-treated H9c2 cells were reversed significantly. Similarly, ferulic acid inhibited H_2_O_2_-induced upregulation of Bax, cleaved-caspase-3, and p38MAPK phosphorylation, and miR-499-5p inhibitor reversed these protective effects significantly (Figures 4(e)–4(i)). Therefore, both ferulic acid treatment and miR-499-5p overexpression inhibited H_2_O_2_-induced cell injury, and miR-499-5p inhibition reversed the protective effect of ferulic acid on H_2_O_2_-induced cell injury. Thus, these data suggested that ferulic acid protects H9c2 cells from H_2_O_2_-induced injury via the miR-499-5p/*p*21 signaling pathway.

### 3.5. Ferulic Acid Attenuates the Isoprenaline-Induced Heart Injury Mouse Model

**Figure 5:**
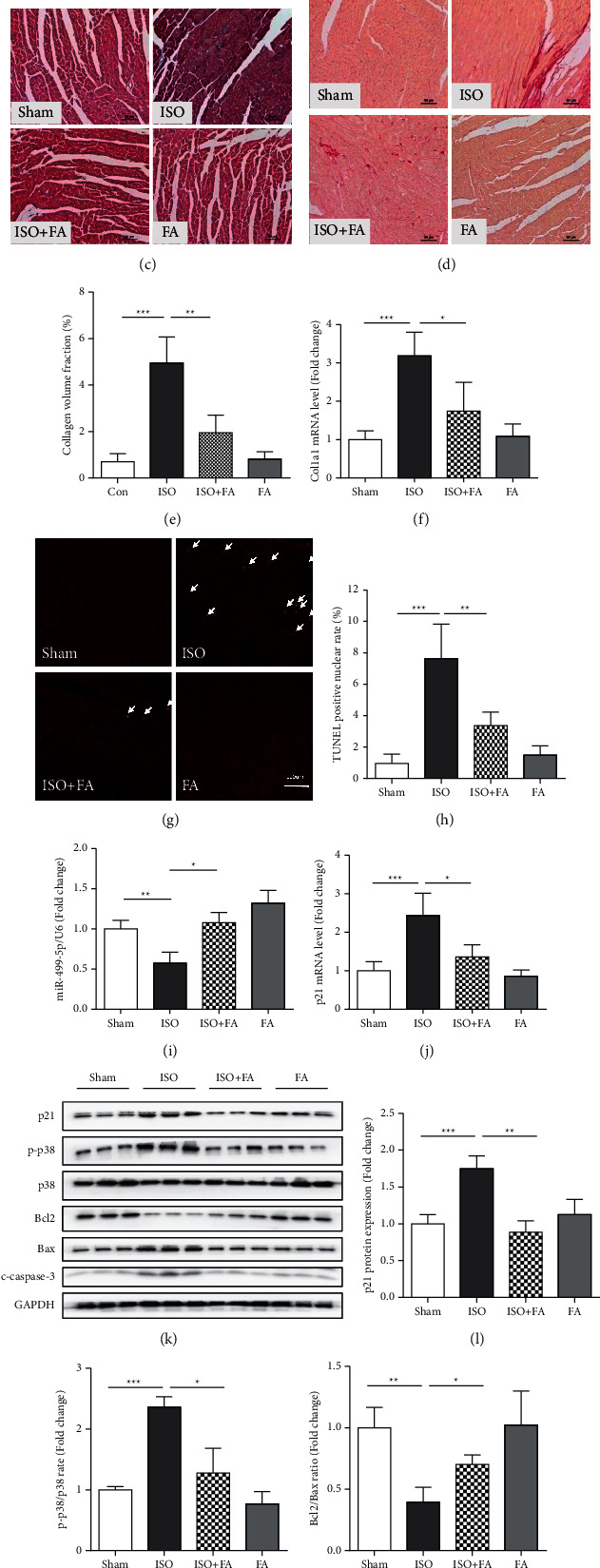
Ferulic acid inhibits isoproterenol-induced oxidative stress and cardiomyocyte apoptosis by regulating the miR-499-5p/*p*21 signaling pathway in vivo. An osmotic pump filled with isoprenaline in saline (30 mg/kg per day) or saline alone was delivered subcutaneously by infusion for 14 days after the operation to insert the pump. Equal volumes of ferulic acid (30 mg/kg/day) or saline were administered to mice daily through oral gavage starting 3 days before the operation and continuing until the end of the experiments. (a), (b) Representative images of mouse hearts and the average heart weight to tibia length ratio (HW/TL) in sham, isoproterenol- (ISO-), isoproterenol + ferulic acid- (ISO + FA-), and ferulic acid- (FA-) treated mice (*n* = 6). (c) Histological analysis of heart sections by H&E staining in sham-, ISO-, ISO + FA-, and FA-treated mice (*n* = 6). (d). (e) Images of Sirius Red and quantitative analysis of heart fibrosis of the heart sections of sham-, ISO-, ISO + FA-, and FA-treated mice (*n* = 6). (f), (g) TUNEL staining and statistical analysis of the TUNEL-positive nucleus rates of heart sections from sham-, ISO-, ISO + FA-, and FA-treated mice (*n* = 6). (h), (i), (j) Real-time quantitative PCR analysis of miR-499-5p, p21, and Col1a1 mRNA levels in the four groups (*n* = 6). (k) Western blot and statistical analysis of p21, p-p38, p38, Bcl-2, Bax, and c-caspase-3 in the four groups of mice (*n* = 6). ^∗^*P* < 0.05, ^∗∗^*P* < 0.01, and ^∗∗∗^*P* < 0.001.

To further verify the findings on cardiomyocytes in vitro, we established an isoproterenol-treated C57BL6 mouse myocardial injury model to investigate the cardioprotective effect of ferulic acid. Subcutaneous infusion of 30 mg/kg/day isoproterenol by osmotic pump for 14 days could induce a mouse heart injury model in vivo. As shown in Figures 5(a) and 5(b), the mouse hearts were enlarged, and the ratio of the heart weight to the tibia length was significantly increased, suggesting that isoprenaline could cause heart hypertrophy in vivo. H&E staining (Figure 5(c)), Sirius Red staining (Figures 5(d) and 5(e)), and Col1a1 mRNA (Figure 5(f)) expression of the heart tissue showed a significant cardiomyocyte size increase and myocardial fibrosis in vivo, suggesting that isoproterenol could cause significant heart remodeling. TUNEL staining (Figures 4(g) and 4(h)) also confirmed that the apoptotic cell rate was upregulated in cardiac tissue, suggesting that isoprenaline causes not only heart hypertrophy but also cardiomyocyte cell death. Interestingly, oral gavage of 30 mg/kg/day ferulic acid alleviated isoprenaline-induced heart injury in vivo.

Further, we analyzed the expression level of miR-499-5p in myocardial tissues by real-time quantitative PCR. Isoprenaline subcutaneous infusion reduced the expression level of miR-499-5p in the mouse heart. However, ferulic acid restored miR-499-5p and p21 expression in isoprenaline-treated mouse hearts (Figures 5(i) and 5(j)). Western blot analysis of mouse heart samples also proved the results from cell experiments. Therefore, ferulic acid can reduce oxidative stress, inflammation, and cell apoptosis and protect heart function in an isoprenaline-induced mouse heart injury model, suggesting that ferulic acid can attenuate isoprenaline-induced heart injury and protect heart function in vivo.

## 4. Discussion

Ferulic acid is a phenolic acid that is commonly found in vegetables, cereals, and traditional Chinese medicines [18]. It was reported that one of the most important properties of ferulic acid is its antioxidant activity, which mainly depends on the hydroxyl and methoxy groups of the benzene ring [19]. S. Chowdhury et al. reported that ferulic acid is more easily absorbed by the body and stays in the blood for a longer time than other phenolic acids. Therefore, ferulic acid is considered to be a good antioxidant [20]. Ferulic acid has lower toxicity and has been widely used in pharmaceuticals and foods. Previous studies have found that, in randomized clinical trials, ferulic acid can improve blood lipid status, oxidative stress, and inflammation in patients with hyperlipidemia [21]. In addition, there is evidence that ferulic acid can quench ROS to exert its antioxidant properties [22]. Oxidative stress is crucial in the occurrence and development of a plethora of diseases, including atherosclerosis [23], chronic obstructive pulmonary disease (COPD) [24], and Alzheimer's disease (AD) [25]. Here, we explored the role of ferulic acid in oxidative stress-induced cardiomyocyte injury. Our results confirmed that both H_2_O_2_ and isoprenaline could cause oxidative stress and inflammation in cardiomyocytes, and ferulic acid could inhibit both H_2_O_2_- and isoprenaline-induced oxidative stress and inflammation in vitro and in vivo.

To further explore the effects of ferulic acid on oxidative stress-induced cardiomyocyte injury, we also analyzed H_2_O_2_- and isoprenaline-induced apoptosis with or without ferulic acid treatment. Some studies found that ferulic acid significantly reduced diabetes-induced kidney damage by inhibiting apoptosis, inflammation, and autophagy defects [20]. The administration of ferulic acid significantly inhibited the colonic apoptosis induced by trinitrobenzenesulfonic acid (TNBS) [26]. R. Sahu et al. showed that wheat phenolics, including ferulic acid, inhibited cardiotoxicity caused by doxorubicin by inhibiting oxidative stress and cardiomyocyte apoptosis [12]. These studies indicate that ferulic acid exerts its biological function through the inhibitory activity on apoptosis. In our study, ferulic acid inhibited both H_2_O_2_- and isoprenaline-induced inflammation and cardiomyocyte apoptosis in vivo and in vitro significantly.

MiRNAs play an important role in the negative regulation of gene expression by pairing with the 3′-UTR of protein-coding gene mRNAs. Many studies have found that miRNAs are involved in the development and progression of many cardiovascular diseases, including heart hypertrophy, myocardial infarction, myocardial ischemia reperfusion, and heart failure [27]. MiR-499-5p is an evolutionarily conserved microRNA encoded by myosin. It is highly expressed in the ventricles [28,29] and plays an important function in cardiomyocytes during ischemia, hypoxia, or oxidative stress [30,31]. A clinical study found that the expression of miR-499-5p increased significantly in the serum of patients with acute myocardial infarction, suggesting that plasma miR-499-5p is a potential biomarker to predict acute myocardial infarction [32,33]. Recent studies have also found that miR-499-5p attenuates mitochondrial fission and apoptosis by downregulating p21 in doxorubicin-mediated cardiotoxicity [34]. In this study, we found that miR-499-5p expression was decreased after treatment with H_2_O_2_ or isoprenaline, which in turn promoted p21 expression and proapoptotic signal transduction in vivo and in vitro. Ferulic acid could promote miR-499-5p expression and inhibit cell apoptosis. The protective effect was abolished by the miR-499-5p inhibitor. Our study suggested that ferulic acid may be an effective drug for the treatment of oxidative stress-induced cardiomyocyte injury by regulating the miR-499-5p/*p*21 signaling pathway.

In this study, we demonstrated that ferulic acid suppresses H_2_O_2_- or isoprenaline-induced cardiomyocyte apoptosis in vivo and in vitro. Ferulic acid treatment effectively protects the heart from oxidative stress and inflammation. Therefore, our findings may implicate novel therapeutic strategies for oxidative stress and inflammation-related heart diseases, including heart hypertrophy, myocardial infarction, and myocardial ischemia reperfusion.

## 5. Conclusions

In summary, we demonstrated that ferulic acid could suppress oxidative stress-induced cardiomyocyte injury by regulating the miR-499-5p/*p*21 signaling pathway. These findings provide insight into the mechanisms by which ferulic acid acts as a potential therapeutic prescription for the treatment of oxidative stress-induced cardiovascular diseases, including heart hypertrophy, myocardial infarction, and myocardial ischemia reperfusion.

## Figures and Tables

**Table 1:** Sequences for cell transfection and primers for RT–PCR.

Group name	Sequences
miR-499-5p mimic	Sense: 5′-UUAAGACUUGCAGUGAUGUUU-3′
	Antisense: 5′-AAACAUCACUGCAAGUCUUAA-3′
Negative control (nc)	Sense: 5′-UCACAACCUCCUAGAAAGAGUAGA-3′
	Antisense: 5′-UCUACUCUUUCUAGGAGGUUGUGA-3′
miR-499-5p inhibitor	5′-AAACAUCACUGCAAGUCUUAA-3′
Negative control of miRNA inhibitor (nci)	5′-CAGUACUUUUGUGUAGUACAA-3′
Rattus-miR-499-5p	RT primer: 5′-GTCGTATCCAGTGCAGGGTCCGAGGTATTCGCACTGGATACGACAAACATC-3′ PCR forward primer: 5′-CGTCCGATTAAGACTTGCAGT-3′
U6	RT primer: 5′-GTCGTATCCAGTGCAGGGTCCGAGGTATTCGCACTGGATACGACAAAATATG-3′ PCR forward primer: 5′-GCGCGTCGTGAAGCGTTC-3′
Universal PCR reverse primer for miRNA and U6	5′-GTGCAGGGTCCGAGGT-3'
Mus-p21 PCR primers	5'-GTACTTCCTCTGCCCTGCTG-3′ 5′- AGAGTGCAAGACAGCGACAA-3'
Mus-col1a1 PCR primers	5'-GAGCGGAGAGTACTGGATCG-3′ 5′-GTTCGGGCTGATGTACCAGT-3′

## Data Availability

All the data needed to evaluate the conclusions in the paper are present in the paper. Additional data related to this paper may be requested from the authors.
